# Interactive Association of XRCC1, XRCC2, XRCC3, and TP53 Gene Polymorphisms With Gastrointestinal Cancer Risk: Insights From a Hospital-Based Case-Control Study

**DOI:** 10.7759/cureus.61921

**Published:** 2024-06-07

**Authors:** Kailas D Datkhile, Rashmi Gudur, Madhavi N Patil, Anand Gudur

**Affiliations:** 1 Department of Molecular Biology and Genetics, Krishna Vishwa Vidyapeeth (Deemed to be University), Karad, IND; 2 Department of Oncology, Krishna Vishwa Vidyapeeth (Deemed to be University), Karad, IND; 3 Department of Oncology, Krishna Charitable Hospital, Karad, IND

**Keywords:** gastrointestinal cancer, xrcc3, genetic polymorphism, tp53, xrcc2, xrcc1

## Abstract

Background and aim: Gastrointestinal (GI) cancer presents a significant worldwide health burden, influenced by a combination of genetic and environmental factors. This study endeavors to explore the combined effects of the *XRCC1*, *XRCC2*, *XRCC3*, and *TP53* genes that contribute to the heightened risk of GI cancer, shedding light on their combined influence on cancer susceptibility.

Materials and methods: A total of 200 histologically confirmed cases of GI cancer and an equal number of controls were selected to examine genetic polymorphisms within the *XRCC1*, *XRCC2*, *XRCC3*, and *TP53* genes using the polymerase chain reaction-based restriction fragment length polymorphism (PCR-RFLP). Odds ratios (OR) with 95% confidence intervals (CI) were calculated to assess the association of these polymorphisms with GI cancer susceptibility, with statistical significance (p ≤ 0.05).

Results: Logistic regression analysis confirmed strong evidence of synergistic interactions among specific variant genotypes. Notably, combinations such as heterozygous Arg/Ser+Ser/Ser genotype of *TP53* Arg249Ser polymorphism with Arg/Trp+Trp/Trp genotype of *XRCC1* Arg194Trp polymorphism (OR=2.64; 95% CI: 1.35-5.18; p=0.004), Arg/Gln+Gln/Gln genotype of *XRCC1* at codon 399 (OR=5.04; 95% CI: 2.81-9.05; p=0.0001), Arg/His and His/His genotypes of *XRCC2* Arg188His (OR=2.16; 95% CI: 1.06-4.39; p<0.032), and Thr/Met+Met/Met genotype of *XRCC3* Thr242Met (OR=3.48; 95% CI: 1.79-6.77; p=0.0002) showed significant associations with GI cancer risk in the study population.

Conclusions: The findings indicate a notable association between the combined effect of heterozygous variant genotypes of *TP53* and variant genotypes of *XRCC1*, *XRCC2*, and *XRCC3* on GI cancer risk. However, further research with a larger sample size and broad single nucleotide polymorphism (SNP) spectra is necessary to understand the interaction between genetic variations and environmental factors influencing GI cancer susceptibility.

## Introduction

Gastrointestinal (GI) cancer is one of the most pressing global health concerns and the fourth leading cause of cancer-related deaths worldwide. In 2022, there were 5.1 million new cases and 3.4 million deaths from GI cancer, with projections to increase by 60% to 75% in the next two decades [1]. Understanding the presentation and progression of GI cancer requires a comprehensive grasp of the complex interplay between genetic and environmental factors, which leads to the accumulation of genetic alterations disrupting normal cellular functions in individuals. The X-ray cross-complementing (XRCC) genes form vital components of the DNA repair mechanism, addressing both single-strand breaks (SSBs) and double-strand breaks (DSBs), thereby contributing to maintaining genomic stability. *XRCC1, XRCC2, *and *XRCC3* are important regulators of the base excision repair (BER) pathway, where *XRCC1* is an integral part of SSB repair, *XRCC2* contributes to DSB repair, and *XRCC3* is connected to double-strand breaks via the homologous recombination repair (HRR) pathway. Their roles are deemed essential for cancer prevention. The *XRCC* genes are highly polymorphic, where three polymorphisms, including Arg>Trp at codon-194 in exon-6 (rs1799782), Arg>His at codon-280 in exon-9 (rs25489), and Arg>Gln at codon-399 in exon-10 (rs25487), are most commonly studied polymorphisms of the *XRCC1* gene [2].

Additionally, the commonly studied polymorphisms associated with cancer susceptibility include the substitution of histidine for arginine at codon 188 of exon 3 of the *XRCC2* gene (rs3218536) and the transition of C to T at codon 241 of exon 7 of the *XRCC3* gene (rs5861539), resulting in the substitution of threonine with methionine [3]. The tumor suppressor (*TP53*) genes are recognized for their involvement in DNA repair mechanisms, cell cycle regulation, maintenance of genomic stability, and other essential cell signaling functions, including the induction of apoptosis. Similarly, *TP53* exhibits high polymorphism, with 13 identified single nucleotide polymorphisms (SNPs) to date, the most commonly studied being Arg72Pro at codon 72 in exon-4 (rs1042522), characterized by a G to C transversion, and Arg249Ser at codon 249 in exon-7 (rs28934571), where a base substitution of G to T at the third position results in the replacement of arginine with serine amino acid [4]. An individual's susceptibility to cancer development is significantly influenced by their genetic background.

The role of genetic polymorphisms in key DNA repair genes such as *XRCC1, XRCC2*, and *XRCC3*, along with their respective SNPs, has been extensively studied for their association with cancer risk across different types of cancers, such as lung [5], breast [6], and cervical cancer [7]. Similarly, the tumor suppressor *TP53* gene with polymorphisms at codon 72 and codon 249 is recognized for its association with lung [8], ovarian [9], and cervical cancer risk [10]. However, the combined effects of polymorphic variants of various SNPs may also be significant in influencing their impact on cancer development across different populations [11]. The combined interactions between SNPs in the *XRCC1*, *XRCC2*, and *XRCC3* genes, along with *TP53* gene polymorphisms, have also been reported and are associated with gastric [12], cervical [13], and breast cancer susceptibility [14,15]. Despite studying multiple polymorphisms in various genes for their association with cancer risk, the understanding of the combined effects of SNP-SNP interactions between these genes, which contribute to an increased risk of GI cancer, remains insufficient. We found limited data on the genetic polymorphisms and interactions between the *XRCC1*, *XRCC2, XRCC3*, and *TP53* genes involved in cancer development, as well as their association with the risk of GI cancer or other carcinomas documented in India.

There is a need to further explore this area to comprehend their cumulative effects on GI carcinogenesis. Therefore, in this hospital-based case-control study, we aimed to assess the impact of SNPs of DNA repair genes including Arg194Trp, C26304T, at exon-5, (rs1799782); Arg399Gln, G28152A at exon-10, (rs25487) of *XRCC1*; Arg188His, G31479A at exon-3, (rs3218536) of *XRCC2, *and Thr241Met, C18067, at exon-7, (rs861539) of *XRCC3* and Arg72Pro at exon 4, (rs1042522) and Arg249Ser at exon-7, (rs28934571) SNPs of tumor suppressor *TP53* gene and their collective association with risk of GI cancer among the population residing in rural Maharashtra, India.

## Materials and methods

Study population

This study involved 200 individuals with clinically confirmed GI cancer and an equal number of healthy controls matched for age and sex, free from any disease. All cases, enrolled immediately after diagnosis at Krishna Hospital and Medical Research Centre between 2018 and 2021, spanned an age range of 20 to 85 years, with a mean age of 59.0 ± 13.32 years (mean ± SD). Controls were matched to cases based on frequency in age groups and sex at the time of enrollment. Trained interviewers collected demographic and clinical data from the participants using a structured questionnaire prepared for this purpose.

Written informed consent was obtained from all eligible cases and controls upon agreeing to participate, following a detailed description of the study. The study protocol (IEC-164/2017-2018) was approved by the Institutional Ethics Committee of Krishna Institute of Medical Sciences for the utilization of human subjects.

Blood sample collection and genomic DNA extraction and purification

After obtaining informed consent, sterile EDTA-containing vacutainers were used to collect 5 milliliters (mL) of whole blood from 200 patients and healthy controls. Genomic DNA extraction from peripheral blood samples was performed using the HipurA® Blood genomic DNA miniprep purification kit (Cat no. MB504-250PR, HiMedia Laboratories, Thane, India), following the manufacturer’s instructions. Subsequently, the purified genomic DNA was utilized for genotyping assays employing polymerase chain reaction (PCR) and restriction fragment length polymorphism (RFLP).

Selection of SNPs and genotyping

The BER genes and tumor suppressor genes are selected in the current study based on reported information from the literature. The polymorphisms of *XRCC1, XRCC2, *and *XRCC3* were identified using the polymerase chain reaction-based restriction fragment length polymorphism (PCR-RFLP) method described previously [16]. Each PCR reaction was carried out separately in a 20 µL volume with 100 nanograms (ng) of purified genomic DNA template, 1X PCR buffer (10 millimolar (mM) Tris-HCl (pH 9.0), 50 mM KCl 1.5 mM MgCl_2_), 0.2 mM each dNTP, 1U Taq DNA polymerase, and 10 picomoles (pmol) primes described earlier [16]. The PCR amplification of a 485 bp codon 194 of *XRCC1 *was carried out at an annealing temperature of 61 °C with 30 cycles of 95 °C - 30 seconds (sec), 61 °C - 30 sec, and 72 °C - 30 sec. The codon 399 of *XRCC1 *(871 bp) amplified with PCR conditions, including initial denaturation at 95 °C for 10 minutes (min) followed by 30 cycles of 95 °C - 20 sec, 56 °C - 30 sec, 72 °C - 20 sec, and final extension at 72 °C - 10 min. The PCR conditions for 290 bp *XRCC2* codon 188 and 455 bp sized codon 241 of* XRCC3 *were annealed at 58 °C and 53 °C for 30 sec, respectively, following 30 cycles of 95 °C - 30 sec, 58 °C - 30 sec, 72 °C - 30 sec, and 72 °C - 10 min). After confirmation of PCR, each PCR product of *XRCC1* exon 6, exon 10, *XRCC2 *exon 3, and *XRCC3* exon 7 was digested overnight at 37 °C with 1 unit (U) of restriction enzymes PvuII, NciI, HphI, and NlaII, respectively.

Additionally, DNA samples were genotyped for SNPs at *TP53* codon 72 and *TP53 *codon 249 loci using PCR-RFLP based on previous research [16]. The PCR cycling conditions for the amplification of 309 bp of cd-72 of *TP53* involved an initial denaturation at 95 °C for 5 min followed by 30 cycles of 95 °C - 30 sec, 52 °C - 30 sec, 72 °C - 30 sec, and final extension at 72 °C - 10 min. Similarly, for amplifying 286 bp of cd-249 of *TP53*, the PCR cycling conditions involved an initial denaturation at 95 °C for 5 min followed by 30 cycles of 95 °C - 20 sec, 60 °C - 30 sec, 72 °C - 20 sec, and final extension at 72 °C - 10 min. The PCR products were digested with 1 U of BstuI and HaeIII for cd72 and cd249 of *TP53*, respectively. The digestion products were then separated on 2%-3% low electroendosmosis (EEO) agarose gel stained with ethidium bromide at 100 V for 30 min and photographed in a gel documentation system. The validation of PCR-RFLP results was performed by DNA sequencing of randomly selected samples. The PCR products were purified using a PCR purification kit (cat. no. #K0701; Thermo Scientific, Waltham, Massachusetts). The DNA sequencing was carried out using the ABI PRISM 310 Analyzer (PathCare Laboratories Private Limited, Hyderabad, India) by a dideoxy nucleotide chain termination method.

Statistical analysis

The study investigated the association between *XRCC1, XRCC2, XRCC3*, and *TP53* genotypes and the risk of developing GI cancer by calculating the odds ratio (OR) with a 95% confidence interval (CI) through Chi-square analysis. Confirmation of the SNP-SNP interactions was conducted using logistic regression analysis with IBM SPSS Statistics for Windows, Version 20 (Released 2011; IBM Corp., Armonk, New York). A significance level of p ≤ 0.05 was considered indicative of association.

## Results

Evaluating *XRCC1*, *XRCC2*, *XRCC3*, and *TP53* genotype frequencies in GI cancer patients and controls

The frequency distribution analysis of the BER genes (*XRCC1*,* XRCC2*,* XRCC3*) in conjunction with rs1042522, rs28934571 SNPs of the tumor suppressor TP53 gene confirmed notable findings with significant association of homozygous variant (A/A) genotype of *XRCC1 *(Arg399Gln) (OR=4.28; 95% CI: 1.81-10.80; p=0.0009) while the variant (T/T) genotype of *XRCC3* (Thr241Met) demonstrated negative association with GI cancer risk (OR=0.32; 95% CI: 0.11-0.91; p=0.032) within the studied population. Furthermore, the variant Ser/Ser genotype of *TP53* (Arg249Ser) revealed a significant association with GI cancer susceptibility (OR=3.54; 95% CI: 1.90-6.56; p=0.0001) (Table 1).

**Table 1:** Distribution of genotype and allele frequencies of XRCC1, XRCC2, XRCC3, and TP53 polymorphisms in GI cancer patients and healthy individuals *Significant odds ratio (p<0.05); p-value determined based on χ2 Significance: p<0.05 OR: odds ratio; CI: confidence interval; GI: gastrointestinal

Gene	Genotype/Allele	Cases (n=200) (%)	Control (n=200) (%)	OR (95% CI)	P-value
XRCC1, Arg194Trp, cd194, Ex-6, (rs1799782)	Arg/Arg	136 (68.0)	141 (70.5)	1 (Reference)	
	Arg/Trp	39 (19.5)	35 (17.5)	1.15 (0.69-1.93)	0.581
	Trp/Trp	25 (12.5)	24 (12.0)	1.08 (0.58-1.98)	0.804
	Arg allele	311 (77.75)	317 (79.25	1 (Reference)	
	Trp allele	89 (22.25)	83 (20.75)	1.09 (0.77-1.53)	0.605
XRCC1, G28152A, Arg399Gln, cd-399, Ex-10, (rs25487)	Arg/Arg	79 (39.5)	123 (61.5)	1 (Reference)	
	Arg/Gln	99 (49.5)	69 (34.5)	2.23 (1.47-3.39)	0.0002*
	Gln/Gln	22 (11.0)	8 (4.0)	4.28 (1.81-10.80)	0.0009*
	Arg allele	257 (64.25)	315 (78.75)	1 (Reference)	
	Gln allele	143 (35.75)	85 (21.25)	2.06 (1.50-2.82)	<0.0001*
XRCC2, Arg188His, cd188, exon-3, (rs3218536)	Arg/Arg	156 (78.0)	151 (75.5)	1 (Reference)	
	Arg/His	39 (19.5)	40 (20.0)	0.94 (0.57-1.54)	0.818
	His/His	5 (2.5)	9 (4.5)	0.53 (0.17-1.64)	0.275
	Arg allele	351 (87.75)	342 (85.5)	1 (Reference)	
	His allele	49 (12.25)	58 (14.5)	0.82 (0.54-1.23)	0.350
XRCC3, Thr241Met, cd-241, exon-7, (rs861539)	Thr/Thr	124 (62.0)	138 (69.0)	1 (Reference)	
	Thr/Met	71 (35.5)	45 (22.5)	1.75 (1.12-2.74)	0.013*
	Met/Met	5 (2.5)	17 (16.5)	0.32 (0.11-0.91)	0.032*
	Thr allele	319 (79.75)	321 (80.25)	1 (Reference)	
	Met allele	81 (20.25)	79 (19.75)	1.03 (0.72-1.45)	0.859
TP53, Arg72Pro, cd72, Ex-4, (rs1042522)	Arg/Arg	48 (24.0)	51 (25.5)	1 (Reference)	
	Arg/Pro	93 (46.5)	65 (32.5)	1.52 (0.91-2.52)	0.104
	Pro/Pro	59 (29.5)	84 (42.0)	0.74 (0.44-1.25)	0.266
	Arg allele	189 (47.25)	167 (41.75)	1 (Reference)	
	Pro allele	211 (52.75)	233 (58.25)	0.80 (0.60-1.05)	0.117
TP53, Arg249Ser, cd249, Ex-7, (rs28934571)	Arg/Arg	100 (50.0)	140 (70.0)	1 (Reference)	
	Arg/Ser	57 (28.5)	43 (21.5)	1.85 (1.15-2.97)	0.010*
	Ser/Ser	43 (21.5)	17 (8.5)	3.54 (1.90-6.56)	0.0001*
	Arg allele	257 (64.25)	323 (80.75)	1 (Reference)	
	Ser allele	143 (35.75)	77 (19.25)	2.33 (1.69-3.22)	<0.0001*

In order to explore the association of polymorphisms, we investigated the relationship between SNPs of the* XRCC1, XRCC2, XRCC3*, and *TP53* genes using a recessive genotype model. Notably, *XRCC1* (Arg399Gln) and *TP53* (Arg249Ser) exhibited significant negative association with GI cancer risk (OR=0.40; 95% CI: 0.27-0.61; p<0.0001 and OR=0.42; 95% CI: 0.28-0.64; p=0.0001, respectively), as shown in Table 2.

**Table 2:** Association between GI cancer risk and genetic variants of XRCC1, XRCC2, XRCC3, and TP53 genes in a recessive model. *Significant odds ratio (p<0.05); p-value determined based on χ2 Significance: p<0.05 OR: odds ratio; CI: confidence interval; GI: gastrointestinal

Genes	Genotype	Cases (n=200) (%)	Control (n=200)(%)	OR (95% CI)	P-value
XRCC1 Arg194Trp (rs1799782)	Trp/Trp+Arg/Trp	64 (32.0)	59 (29.5)	1 (Reference)	
	Arg/Arg	136 (68.0)	141 (70.5)	0.88 (0.58-1.36)	0.588
XRCC1 Arg399Gln (rs25487)	Gln/Gln/Arg/Gln	121 (60.5)	77 (38.5)	1 (Reference)	
	Arg/Arg	79 (39.5)	123 (61.5)	0.40 (0.27-0.61)	<0.0001*
XRCC2 Arg188His (rs3218536)	His/His+Arg/His	44 (22.0)	49 (24.5)	1 (Reference)	
	Arg/Arg	156 (78.0)	151 (75.5)	1.15 (0.72-1.83)	0.554
XRCC3 Thr241Met (rs861539)	Met/Met+Thr/Met	76 (38.0)	62 (31.0)	1 (Reference)	
	Thr/Thr	124 (62.0)	138 (69.0)	0.73 (0.48-1.10)	0.141
TP53 Arg72Pro (rs1042522)	Pro/Pro+Arg/Pro	152 (76.0)	149 (74.5)	1 (Reference)	
	Arg/Arg	48 (24.0)	51 (25.5)	0.92 (0.58-1.45)	0.728
TP53 Arg249Ser (rs28934571)	Ser/Ser+Arg/Ser	100 (50.0)	60 (30.0)	1 (Reference)	
	Arg/Arg	100 (50.0)	140 (70.0)	0.42 (0.28-0.64)	0.0001*

The dominant genotype model further supported these findings, revealing* XRCC1* (Arg399Gln) with decreased GI cancer risk (OR=0.33; 95% CI: 0.14-0.77; p=0.010). The evaluation of the dominant genotype model showed that *XRCC3 *(Thr241Met) showed a significant positive association (OR=3.62; 95% CI: 1.30-10.02; p=0.013) with GI cancer risk in the studied population. In our analysis of *TP53* gene polymorphisms and GI cancer risk using dominant genotype model, *TP53* (Arg72Pro) exhibited a significant positive association (OR=1.73; 95% CI: 1.14-2.61; p=0.009), whereas the rs28934571 SNP of *TP53* (Arg249Ser) showed negative association (OR=0.33; 95% CI: 0.18-0.61; p=0.0004) with GI cancer risk (Table 3).

**Table 3:** Association between GI cancer risk and genetic variants of XRCC1, XRCC2, XRCC3, and TP53 genes in a dominant model. *Significant odds ratio (p<0.05); p-value determined based on χ2 Significance: p<0.05 OR: odds ratio; CI: confidence interval; GI: gastrointestinal

Genes	Genotype	Cases (n=200) (%)	Control (n=200) (%)	OR (95% CI)	P-value
XRCC1 Arg194Trp rs1799782	Trp/Trp	25 (12.5)	24 (12.0)	1 (Reference)	
	Arg/Trp+Arg/Arg	175 (87.5)	176 (88.0)	0.95 (0.52-1.73)	0.878
XRCC1 Arg399Gln rs25487	Gln/Gln	22 (11.0)	8 (4.0)	1 (Reference)	
	Arg/Gln+Arg/Arg	178 (89.0)	192 (96.0)	0.33 (0.14-0.77)	0.010*
XRCC2 Arg188His rs3218536	His/His	5 (2.5)	9 (4.5)	1 (Reference)	
	Arg/His+Arg/Arg	195 (97.5)	191 (95.5)	1.83 (0.60-5.58)	0.283
XRCC3 Thr241Met rs861539	Met/Met	5 (2.5)	17 (8.5)	1 (Reference)	
	Thr/Met+Thr/Thr	195 (97.5)	183 (91.5)	3.62 (1.30-10.02)	0.013*
TP53 Arg72Pro rs1042522	Pro/Pro	59 (29.5)	84 (42.0)	1 (Reference)	
	Arg/Pro+Arg/Arg	141 (70.5)	116 (58.0)	1.73 (1.14-2.61)	0.009*
TP53 Arg249Ser rs28934571	Ser/Ser	43 (21.5)	17 (8.5)	1 (Reference)	
	Arg/Ser+Arg/Arg	157 (78.5)	183 (91.5)	0.33 (0.18-0.61)	0.0004*

Investigating the combined effects of *XRCC1, XRCC2, XRCC3*, and *TP53 *gene polymorphisms with GI cancer risk

The combined effects of polymorphism of *XRCC1, XRCC2, *and *XRCC3*, along with *TP53* gene polymorphism, were investigated for their association with GI cancer risk in the rural population of Maharashtra. Specifically, this analysis includes the evaluation of Arginine72Proline and Arginine249Serine polymorphism of *TP53* and their association with BER gene polymorphisms. The findings of the analysis revealed that the combination of variant Gln/Gln genotype of *XRCC1 *Arg399Gln and variant *TP53* Arg72Pro genotype increased the risk of GI cancer with 2.80-fold with OR=2.80; 95% CI: 1.47-5.35; p=0.001.

Moreover, a significant association was observed between the combination of Arg/Arg genotype of Arg399Gln polymorphism of *XRCC1* and Arg/Pro+Pro/Pro genotype of TP53 with an OR of 3.01 (95% CI: 1.35-7.15; p=0.007) in relation to GI cancer risk among the studied population. On the other hand, the polymorphism of Arg194Trp of *XRCC1*, Arg188His of *XRCC2*, and Thr241Met of *XRCC3* in combination with Arg72Pro polymorphism of TP53 did not show an increased risk of GI cancer (p>0.05), according to Table 4.

**Table 4:** Distribution and combined effects of XRCC1, XRCC2, and XRCC3 along with codon 72 of TP53 genotype frequencies and their association with GI cancer risk. *Significant odds ratio (p<0.05); p-value determined based on χ2 Significance: p<0.05 OR: odds ratio; CI: confidence interval; GI: gastrointestinal

Gene and Genotype		GI Cancer Group N=200 (%)	Control Group N=200 (%)	Odds Ratio (OR) (95% CI)	P-value
XRCC1 codon-194	TP53 codon-72				
Arg/Arg	Arg/Arg	36 (18.0)	38 (19.0)	1 (Reference)	
Arg/Trp+Trp/Trp	Arg/Arg	100 (50.0)	103 (51.5)	1.02 (0.60-1.74)	0.928
Arg/Arg	Arg/Pro+Pro/Pro	12 (6.0)	11 (5.5)	1.15 (0.45-2.93)	0.767
Arg/Trp+Trp/Trp	Arg/Pro+Pro/Pro	52 (26.0)	48 (24.0)	1.14 (0.62-2.08)	0.662
XRCC1 codon-399	TP53 codon-72				
Arg/Arg	Arg/Arg	19 (9.5)	35 (17.5)	1 (Reference)	
Arg/Gln+Gln/Gln	Arg/Arg	61 (30.5)	88 (44.0)	1.27 (0.66-2.43)	0.459
Arg/Arg	Arg/Pro+Pro/Pro	27 (13.5)	16 (8.0)	3.01 (1.35-7.15)	0.007*
Arg/Gln+Gln/Gln	Arg/Pro+Pro/Pro	93 (46.5)	61 (30.5)	2.80 (1.47-5.35)	0.001*
XRCC2 codon-188	TP53 codon-72				
Arg/Arg	Arg/Arg	34 (17.0)	32 (16.0)	1 (Reference)	
Arg/His+His/His	Arg/Arg	122 (61.0)	119 (59.5)	0.96 (0.55-1.66)	0.897
Arg/Arg	Arg/Pro+Pro/Pro	14 (7.0)	19 (9.5)	0.69 (0.29-1.61)	0.394
Arg/His+His/His	Arg/Pro+Pro/Pro	30 (15.0)	30 (15.0)	0.94 (0.46-1.89)	0.965
XRCC3 codon-241	TP53 codon-72				
Thr/Thr	Arg/Arg	31 (15.5)	38 (19.0)	1 (Reference)	
Thr/Met+Met/Met	Arg/Arg	93 (46.5)	99 (49.5)	1.15 (0.66-2.00)	0.616
Thr/Thr	Arg/Pro+Pro/Pro	16 (8.0)	13 (6.5)	1.50 (0.63-3.60)	0.355
Thr/Met+Met/Met	Arg/Pro+Pro/Pro	60 (30.0)	50 (25.0)	1.47 (0.80-2.69)	0.211

In individuals with a combination of Arg/Trp+Trp/Trp genotype of *XRCC1* Arg194Trp polymorphism and Arg/Ser+Ser/Ser genotype of *TP53* Arg249Ser polymorphism, the risk of GI cancer was found to increase significantly by 2.64 times (OR=2.64; 95% CI: 1.35-5.18; p=0.004). Notably, a statistically significant correlation was observed between the presence of polymorphic Arg/Gln+Gln/Gln genotype of *XRCC1* at codon 399 and Arg/Ser+Ser/Ser genotype of *TP53* at codon 249, showing a substantial increase in GI cancer risk with an OR of 5.04 (95% CI: 2.81-9.05; p=0.0001). Similarly, the combined effects of Arg/His and His/His genotypes of *XRCC2 *Arg188His with Arg/Ser and Ser/Ser genotypes of *TP53* Arg249Ser were associated with an increase in the risk of GI cancer (OR=2.16; 95% CI: 1.06-4.39; p<0.032) within the studied population.

Additionally, combinations of the variant Arg/Ser+Ser/Ser genotype of *TP53* Arg249Ser and Thr/Met+Met/Met genotype of *XRCC3* Thr242Met significantly elevated the risk of GI cancer by 3.48-fold in the affected patients (OR=3.48; 95% CI: 1.79-6.77; p=0.0002). The detailed insights of genetic polymorphisms and their combined association of *XRCC1, XRCC2*, and *XRCC3 *with *TP53* codon-249 genotypes with GI cancer risk are presented in Table 5.

**Table 5:** Distribution and combined effects of XRCC1, XRCC2, and XRCC3 along with codon 249 of TP53 genotype frequencies and their association with GI cancer risk. *Significant odds ratio (p<0.05); p-value determined based on χ2 Significance: p<0.05 OR: odds ratio; CI: confidence interval; GI: gastrointestinal

Gene and Genotype		GI Cancer Group N=200: n (%)	Control Group N=200: n (%)	Odds Ratio (OR) 95% CI	P-value
XRCC1 codon-194	TP53 codon-249				
Arg/Arg	Arg/Arg	66 (33.0)	99 (49.5)	1 (Reference)	
Arg/Trp+Trp/Trp	Arg/Arg	70 (35.0)	42 (21.0)	2.50 (1.52-4.09)	0.0003*
Arg/Arg	Arg/Ser+Ser/Ser	34 (17.0)	42 (21.0)	1.21 (0.70-2.10)	0.488
Arg/Trp+Trp/Trp	Arg/Ser+Ser/Ser	30 (15.0)	17 (8.5)	2.64 (1.35-5.18)	0.004*
XRCC1 codon-399	TP53 codon-249				
Arg/Arg	Arg/Arg	45 (22.5)	86	1 (Reference)	
Arg/Gln+Gln/Gln	Arg/Arg	34 (17.0)	35 (17.5)	1.85 (1.02-3.36)	0.041*
Arg/Arg	Arg/Ser+Ser/Ser	55 (27.5)	54 (27.5)	1.94 (1.15-3.27)	0.012*
Arg/Gln+Gln/Gln	Arg/Ser+Ser/Ser	66 (33.0)	25 (12.5)	5.04 (2.81-9.05)	0.0001*
XRCC2 codon-188	TP53 codon-249				
Arg/Arg	Arg/Arg	79 (39.5)	107 (53.5)	1 (Reference)	
Arg/His+His/His	Arg/Arg	77 (38.5)	44 (22.0)	2.37 (1.48-3.79)	0.003*
Arg/Arg	Arg/Ser+Ser/Ser	20 (10.0)	34 (17.0)	0.79 (0.42-1.48)	0.475
Arg/His+His/His	Arg/Ser+Ser/Ser	24 (12.0)	15 (7.5)	2.16 (1.06-4.39)	0.032*
XRCC3 codon-241	TP53 codon-249				
Thr/Thr	Arg/Arg	65 (32.5)	98 (49.0)	1 (Reference)	
Thr/Met+Met/Met	Arg/Arg	59 (29.5)	43 (21.5)	2.06 (1.25-3.42)	0.004*
Thr/Thr	Arg/Ser+Ser/Ser	39 (19.5)	43 (21.5)	1.36 (0.80-2.33)	0.251
Thr/Met+Met/Met	Arg/Ser+Ser/Ser	37 (18.5)	16 (8.0)	3.48 (1.79-6.77)	0.0002*

## Discussion

Recent advancements in cancer genetics have deepened our understanding of the significance of SNPs, which serve as decisive signatures for assessing an individual's susceptibility to cancer. Numerous studies have emphasized the significance of SNPs in various pathway genes, elucidating their involvement in the carcinogenesis process. Among these pathways, BER stands out as a crucial mechanism in DNA repair, with the *XRCC1*, *XRCC2*, and *XRCC3* genes being extensively studied for their SNPs. Particularly, the Arg194Trp and Arg399Gln variants of *XRCC1*, the Arg188His variant of *XRCC2*, and the Thr241Met variant of *XRCC3* have been studied for their associations with various types of cancer [5-7]. Similarly, the SNPs Arg72Pro and Arg249Ser in *TP53* have been frequently investigated for their correlation with cancer risk [8-10]. However, there is a dearth of literature exploring the combined effects of genetic polymorphisms in the *XRCC1*, *XRCC2*, *XRCC3*, and *TP53* genes for their involvement in cancer risk.

In this regard, the retrospective analysis of the current study revealed a significant association between the *XRCC1* 399Gln variant genotype and GI cancer risk, aligning with previous research in Chinese [17] and Turkish populations [12]. Several epidemiological studies have shown a positive correlation between the *XRCC1* 399Gln polymorphism and colorectal [18] and hepatocellular carcinoma [19]. Conversely, some studies have reported no association between the 399Gln variant genotype and GI cancer risk [20,21]. In our investigation of the *XRCC2* Arg188His and *XRCC3* Thr241Met polymorphisms and their role in cancer development, we noted no association with the *XRCC2* polymorphism, while *the XRCC3* polymorphism exhibited a negative association, indicating a protective effect against GI cancer risk in the studied population. These findings were in accordance with previous Indian studies that reported no association of *XRCC2* Arg188His polymorphism with cervical [22], nasopharyngeal [23], or breast cancers [24]. Similarly, the rs1042522 and rs28934571 SNPs of *TP53* were comprehensively studied in relation to GI cancer risk [25]. However, others have reported conflicting results or found no association [26].

Based on the genotype frequency of *TP53* (rs1042522), our study found no obvious association between the Arg72Pro polymorphism and GI cancer risk, contrary to the findings in other Indian studies [27,28]. The major finding of this study indicates that the polymorphism in the Ser/Ser allele of codon 249 of exon-7 of *TP53* (rs28934571) contributes to a 3.54-fold increased risk for GI cancer in the study population, which is supported by similar findings with strong association between the 249(Ser) *TP53* polymorphism in colorectal [29], hepatocellular [30], and cervical cancer risk [31]. There is limited literature available regarding the combined effects of the SNP-SNP interaction between genetic polymorphisms of the *XRCC1*, *XRCC2*, *XRCC3*, and *TP53* genes in breast [14,15,32] and cervical cancer susceptibility [13].

In our study, *XRCC1* Arg399Gln significantly correlated with GI cancer risk, while *XRCC3* Thr241Met showed a negative association. However, the interaction of *XRCC1* Arg399Gln and *TP53* Arg72Pro polymorphisms did not show an association with gastric cancer [12]. The *XRCC1* Arg194Trp and *XRCC2* Arg188His showed no significant association with GI cancer. Similarly, *TP53* Arg72Pro did not increase GI cancer risk, but both homozygous and heterozygous Arg249Ser variants were associated with GI cancer risk. Additionally, our study found significant combined effects of *TP53* Arg249Ser variants with *XRCC1* Arg194Trp and Arg399Gln, *XRCC2* Arg188His, and *XRCC3* Thr241Met, indicating increased GI cancer risk, underscoring the importance of the SNP-SNP interactions in determining cancer susceptibility within the studied population.

In this study, we found that the combination of heterozygote Arg72Pro mutant genotype of *TP53* with variant genotypes of *XRCC1* (Arg194Trp, Arg399Gln), *XRCC2* (Arg188His), and *XRCC3* (Thr241Met) did not correlate with GI cancer risk. However, it is important to note that our study had certain limitations, including a small sample size, a hospital-based case-control study design, and potential selection bias due to the limited number of SNPs considered. Further research with a larger sample size is necessary to elucidate interactions between genetic and environmental factors and the risk of GI cancer in the studied population.

## Conclusions

In summary, our study concludes that the combination of the heterozygous variant genotypes of *TP53* with Arg249Ser polymorphism with variant genotypes of *XRCC1*, *XRCC2*, and *XRCC3* was significantly associated with GI cancer risk in the Maharashtrian population. The current findings lack a clear mechanistic explanation, prompting the need for further confirmation in larger populations to elucidate this aspect. Validating our findings in larger cohorts will help to elucidate the collective impacts of SNPs in the *XRCC1*, *XRCC2*, *XRCC3*, and *TP53* genes on GI cancer risk.
